# Age-related and gender-stratified differences in the association between high triglyceride and risk of hyperuricemia

**DOI:** 10.1186/s12944-019-1077-5

**Published:** 2019-07-04

**Authors:** Lei Zhang, Qilin Wan, Yuemin Zhou, Jing Xu, Chengyun Yan, Yuanyuan Ma, Minglong Xu, Ruili He, Yanming Li, Xiaoming Zhong, Guanchang Cheng, Yuquan Lu

**Affiliations:** 1 Department of Cardiology, Huaihe Hospital of Henan University, No. 8 Baobei Road, Kaifeng, 475000 China; 2 International Joint Research Laboratory for Cell Medical Engineering of Henan, Huaihe Hospital of Henan University, No. 115 Ximen Street, Kaifeng, 475000 China; 3 Department of Physical Examination Center, Huaihe Hospital of Henan University, No. 8 Baobei Road, Kaifeng, 475000 China

**Keywords:** Hyperuricemia, Triglyceride, Risk, Age-related

## Abstract

**Background:**

Elevated serum uric acid is commonly associated with high triglyceride. However, the relation of triglyceride and hyperuricemia in different gender and age groups is currently not well understood. This study aimed to evaluate age- and gender-related association of high triglyceride with hyperuricemia in a subgroup of Chinese population.

**Methods:**

We retrospectively analyzed physical examination data of 24,438 subjects (12,557 men and 11,881 women) in Kaifeng, China. The alanine aminotransferase, γ-glutamyl transpeptidase, serum creatinine, blood urea nitrogen, total cholesterol, high-density lipoprotein cholesterol, triglyceride and serum uric acid were measured in all subjects. The triglyceride was categorized into < 1.21, 1.21 ~, 1.7 ~, 2.83 ~ and >  5.6 mmol/L subgroups, and odds ratio (OR) and 95% confidence interval (CI) of hyperuricemia were calculated by logistic regression analysis.

**Results:**

Univariate and age-adjusted analyses showed that high triglyceride was positively associated with hyperuricemia (*p* <  0.01). Further age-stratified analysis showed that the positive association was significant in the 20 ~, 30 ~, 40 ~, 50 ~, 60 ~ and 80 ~ age groups in men. In women, no statistically significant was found in 60 ~ and 70 ~ age groups.

**Conclusion:**

High triglyceride is positively associated with hyperuricemia in both men and women, and this association is age-related, especially in women.

## Background

Hyperuricemia is widely considered as a key risk factor for metabolic syndrome, including dyslipidemia, in which hypertriglyceridemia is the most common lipid abnormality [1–6]. High uric acid (UA) levels were associated with increased triglyceridemia, independently of metabolic syndrome [7]. A survey of the prevalence of obesity in China during 2004 to 2008 showed that all rural areas had a low prevalence of obesity except in Henan, probably because of differences in diet [8]. Furthermore, a recent meta-analysis evaluating 302,430 individuals free of known vascular disease at baseline in 68 prospective studies showed that high triglyceride had a strong association with both cardiovascular disease and ischemic stroke [9]. In addition, the secondary prevention of those diseases and antihyperuricemic therapy involve long term and high costs [10–12]. Therefore, if we can identify the link of triglycerides with hyperuricemia in different age groups, more people will benefit.

Liou et al. reported that uric acid aggregated with log value of triglycerides but concluded that metabolic syndrome was not associated with hyperuricemia [13]. However, an Indian study enrolling 121 healthy men suggested that serum uric acid level was a good indicator of the level of triglyceride [14]. At present, little is known whether serum uric acid level and high triglyceride has the similar relationship in Chinese in different age-groups. Therefore, this study aimed to investigate the association of high triglyceride with the risk of hyperuricemia in different genders and age-groups using physical examination data from a hospital-based physical examination center in Kaifeng, China.

## Methods

### Subjects

**Fig. 1:**
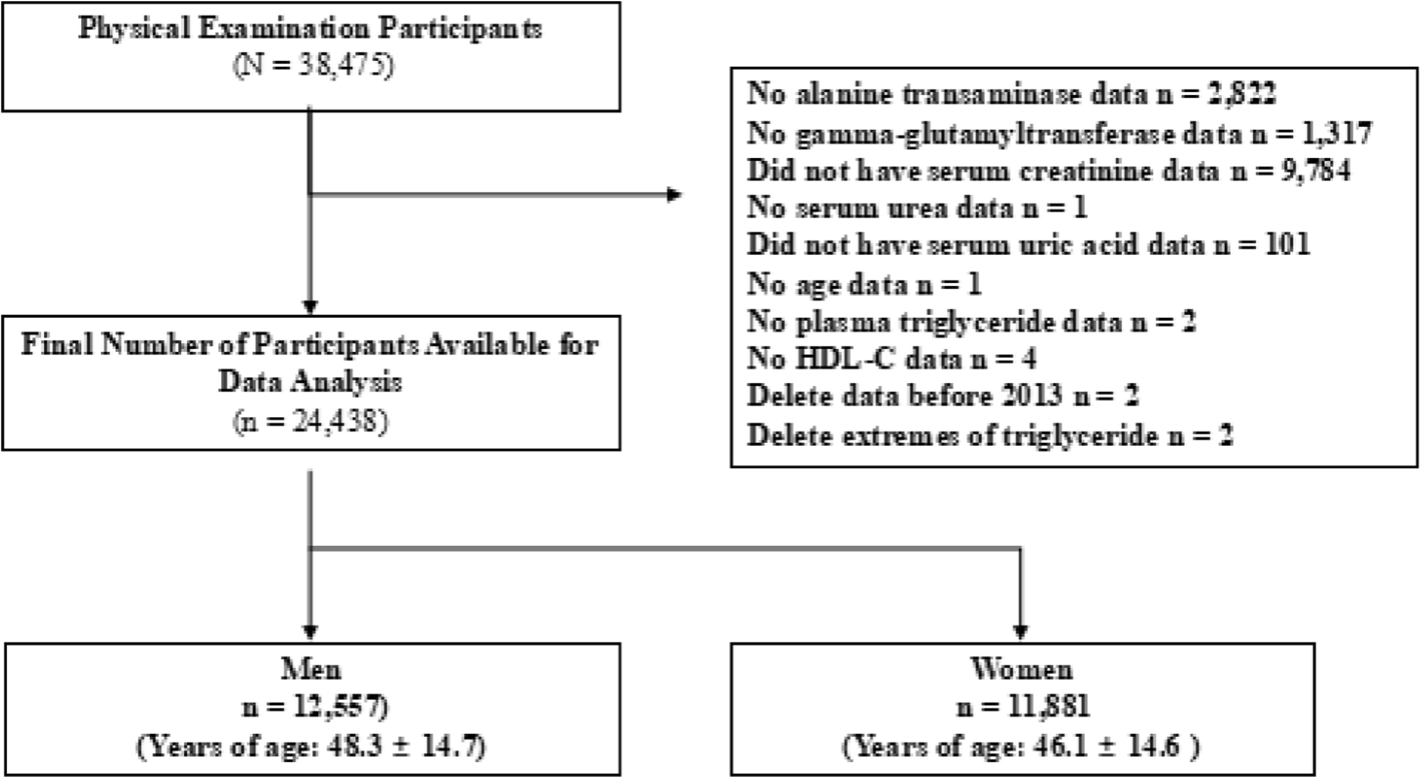
Flow chart of participants

This study was approved by Ethics Committee of Huaihe Hospital of Henan University and all subjected provided written consent. The subjects were participants of physical examination at the Physical Examination Center of Huaihe Hospital of Henan University. Total number of the subjects were 38,475 consecutive participants from 2003 to 2017. After excluding subjects without available data on age, gender, total cholesterol (TC), triglyceride (TG), high-density lipoprotein cholesterol (HDL-C), alanine aminotransferase (ALT), γ-glutamyl transpeptidase (γ-GT), blood urea nitrogen (BUN) and serum creatinine (SCr), a total of 24,438 eligible subjects (12,557 men and 11,881 women) were included in the final analysis (Fig. 1).

### Serum uric acid and lipids measurements

Blood samples from participants undergoing an overnight fasting were collected in the morning and analyzed within an hour in hospital. Blood sample was tested using an autoanalyzer (Model 7600, HITACHI, Japan). Hyperuricemia was defined by the following criteria: men: serum uric acid ≥440 μmol/L, women: serum uric acid ≥360 μmol/L and categorized all the variables as follows: the plasma triglyceride were categorized into < 1.21 (normal), 1.21 ~ (above normal), 1.7 ~ (slightly damaged), 2.83 ~ (moderately damaged) and >  5.6 mmol/L (severely damaged) subgroups, and we combined the slightly, moderately, and severely damaged TG into a Damaged TG group; age was categorized into < 20, 20 ~, 30 ~, 40 ~, 50 ~, 60 ~, 70 ~, and 80 ~ years age subgroups; ALT was dichotomized into normal (≤ 40 U/L) and abnormal (> 40 U/L) subgroups; γ-GT was categorized into low (< 16 U/L), normal (16 ~ 73 U/L) and high (> 73 U/L) subgroups; SCr was categorized into low (men: < 54 mmol/L, women: < 44 mmol/L), normal (men: 54 ~ 106 mmol/L, women: 44 ~ 97 mmol/L) and high (men: > 106 mmol/L, women: > 97 mmol/L) subgroups; BUN was categorized into low (< 2.86 μmol/L), normal (2.86 ~ 7.14 μmol/L) and high (> 7.14 μmol/L) subgroups; TC was categorized into low (< 2.8 mmol/L), normal (2.8 ~ 5.17 mmol/L) and high subgroups (> 5.17 mmol/L); plasma glucose was categorized into low FPG (< 3.9 mmol/L), normal FPG (3.9 ~ mmol/L), impaired FPG (6.1 ~ mmol/L) and diabetic FPG (> 7.0 mmol/L) subgroups; and HDL-C was categorized into low (< 0.9 mmol/L), normal (0.9 ~ 2.19 mmol/L) and high (> 2.19 mmol/L) subgroups.

### Statistical analysis

Statistical analysis was performed using Stata version 13 (StataCorp LLC, TX, USA). The chi-squared test was used to analyze the differences in the distribution of TG categories. Three models with progressive degrees of adjustment were used to evaluate the relationship between hyperuricemia with TG for men and women, separately. Model 1 was a univariate logistic regression model; Model 2 was an age-adjusted logistic regression model, and Model 3 was a multivariate logistic regression model (adjusted for age, ALT, γ-GT, SCr, BUN, TC, FPG, and HDL-C). Multivariable logistic regression analysis were used to calculate age-stratified association of TG and hyperuricemia for both men and women. Odds ratio (OR) and 95% confidence intervals (CI) were estimated in the logistical regression analysis. *p* <  0.05 was considered significant.

## Results

**Fig. 2:**
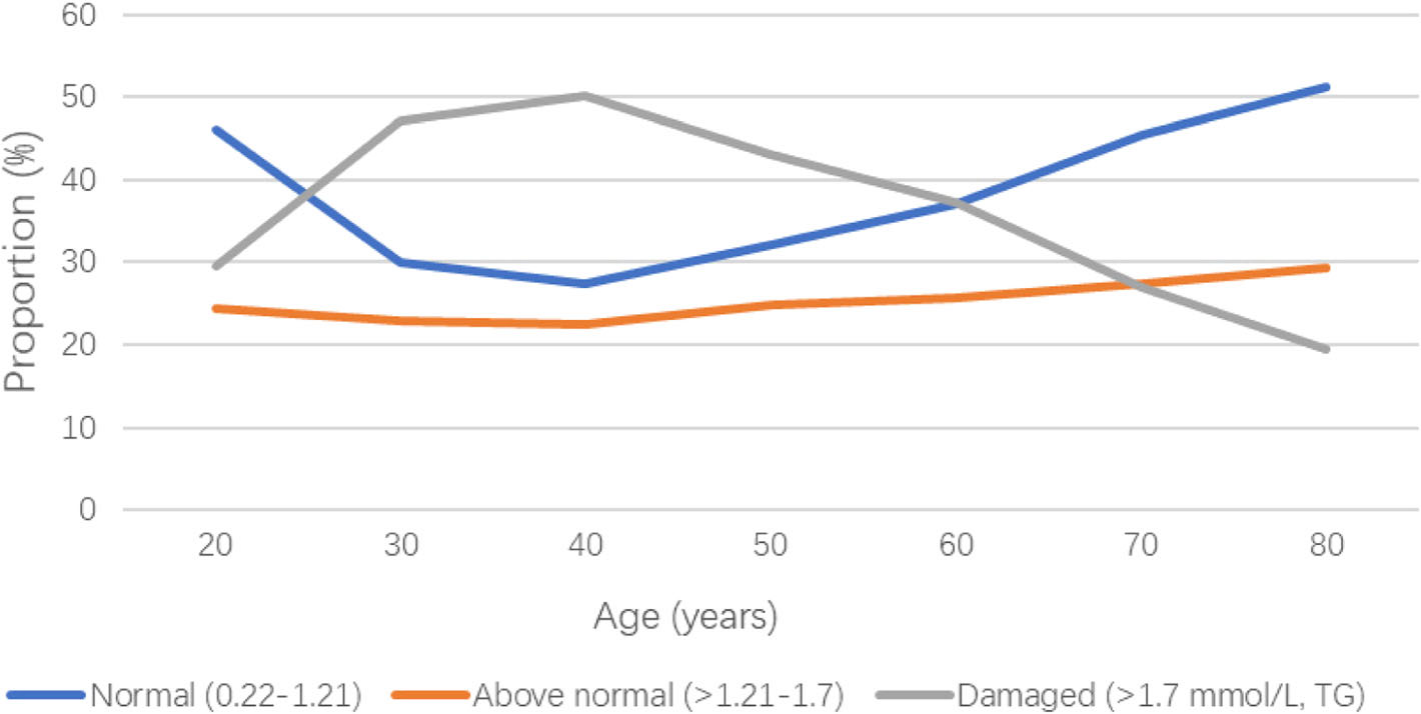
Changes of triglyceride levels with age in men

**Fig. 3:**
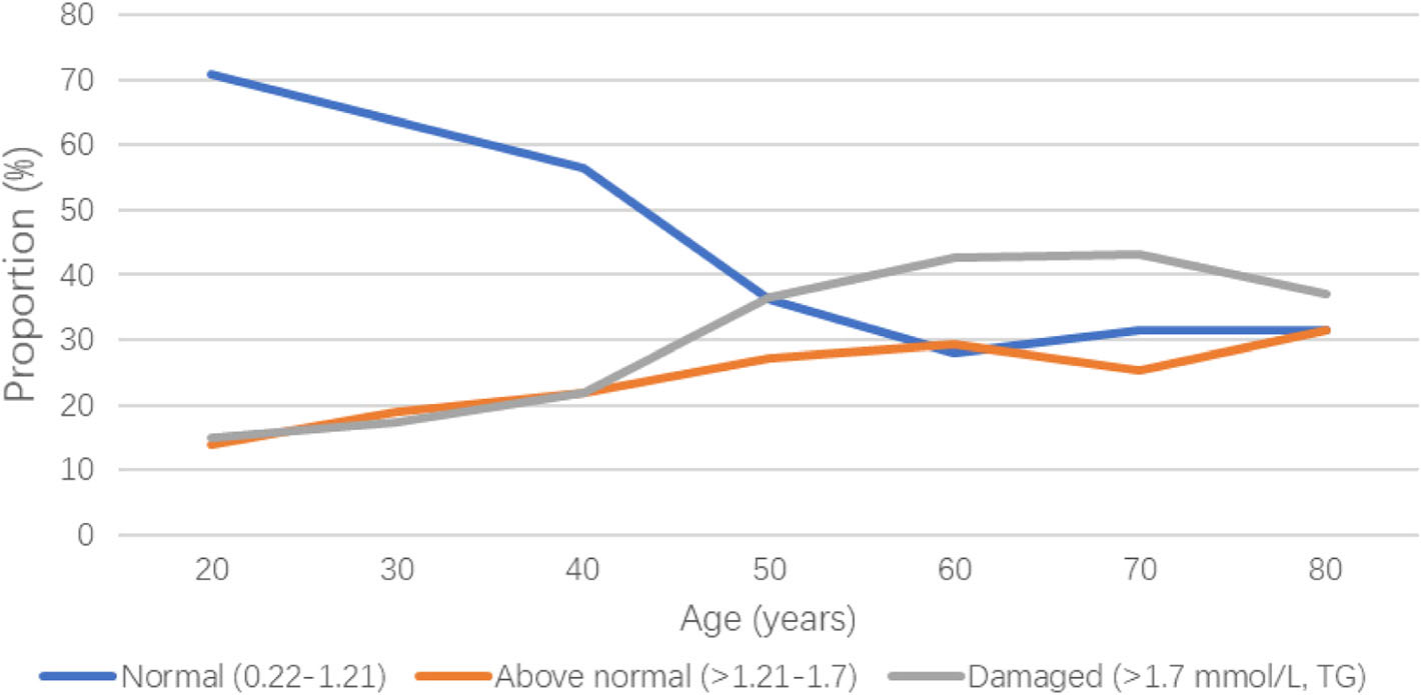
Changes of triglyceride levels with age in women

We observed an increasing trend in the proportion of the Damaged TG group when the age increased from 20 to 40 years; thereafter, the proportion of the Damaged TG group started to decrease with age in men (Fig. 2). There was a growing trend of the proportion of the Damaged TG group among women with a flat plateau from 60 years of age (Fig. 3).

In Table 1, the Damaged TG group (1.7 ~, 2.83 ~ and >  5.6 mmol/L subgroups) had a higher proportion to have high levels of serum ALT, γ-GT, TC and hyperuricemia (all *p* <  0.05), but had a lower proportion to have high level of serum HDL-C (*p* <  0.001) in both genders.

**Table 1:** Characteristics of the participants of physical examinations by fasting plasma triglyceride categories (mmol/L) in men and women

	Men, fasting plasma triglyceride (mmol/L)							Women, fasting plasma triglyceride (mmol/L)						
	No. of participants	< 1.21	1.21 ~	1.7 ~	2.83 ~	> 5.6	*P*-value	No. of participants	< 1.21	1.21 ~	1.7 ~	2.83 ~	> 5.6	*P*-value
No. of participants	12,557	4293 (34.2)	3040 (24.2)	3335 (26.6)	1546 (12.3)	343 (2.7)		11,881	6023 (50.7)	2661 (22.4)	2353 (19.8)	747 (6.3)	97 (0.8)	
Age (years), n (%)														
< 20	43	33 (76.7)	4 (9.3)	5 (11.6)	0 (0.0)	1 (2.3)	< 0.001	22	17 (77.3)	3 (13.6)	2 (9.1)	0 (0.0)	0 (0.0)	< 0.001
20 ~	1325	609 (46.0)	324 (24.5)	244 (18.4)	125 (9.4)	23 (1.7)		1880	1335 (71.0)	264 (14.0)	190 (10.1)	81 (4.3)	10 (0.5)	
30 ~	2348	702 (29.9)	540 (23.0)	669 (28.5)	342 (14.6)	95 (4.1)		2485	1582 (63.7)	473 (19.0)	324 (13.0)	89 (3.6)	17 (0.7)	
40 ~	3133	858 (27.4)	704 (22.5)	938 (29.9)	505 (16.1)	128 (4.1)		2652	1496 (56.4)	578 (21.8)	436 (16.4)	128 (4.8)	14 (0.5)	
50 ~	2735	879 (32.1)	680 (24.9)	764 (27.9)	349 (12.8)	63 (2.3)		2492	904 (36.3)	679 (27.3)	654 (26.2)	224 (9.0)	31 (1.2)	
60 ~	1848	684 (37.0)	474 (25.7)	510 (27.6)	157 (8.5)	23 (1.2)		1509	424 (28.1)	441 (29.2)	480 (31.8)	146 (9.7)	18 (1.2)	
70 ~	848	386 (45.5)	233 (27.5)	166 (19.6)	56 (6.6)	7 (0.8)		679	214 (31.5)	172 (25.3)	222 (32.7)	64 (9.4)	7 (1.0)	
80 ~	277	142 (51.3)	81 (29.2)	39 (14.1)	12 (4.3)	3 (1.1)		162	51 (31.5)	51 (31.5)	45 (27.8)	15 (9.3)	0 (0.0)	
Alanine Aminotransferase (U/L), n (%)														
<= 40	10,864	3974 (36.6)	2694 (24.8)	2812 (25.9)	1165 (10.7)	219 (2.0)	< 0.001	11,289	5856 (51.9)	2514 (22.3)	2172 (19.2)	661 (5.9)	86 (0.8)	< 0.001
> 40	1693	319 (18.8)	346 (20.4)	523 (30.9)	381 (22.5)	124 (7.3)		592	167 (28.2)	147 (24.8)	181 (30.6)	86 (14.5)	11 (1.9)	
γ-Glutamyl Transpeptidase (U/L), n (%)														
< 16	1722	1102 (64.0)	375 (21.8)	200 (11.6)	39 (2.3)	6 (0.4)	< 0.001	6207	4088 (65.9)	1253 (20.2)	716 (11.5)	138 (2.2)	12 (0.2)	< 0.001
16 ~ 73	9724	3077 (31.6)	2509 (25.8)	2766 (28.5)	1168 (12.0)	204 (2.1)		5488	1892 (34.5)	1364 (24.9)	1578 (28.8)	575 (10.5)	79 (1.4)	
> 73	1111	114 (10.3)	156 (14.0)	369 (33.2)	339 (30.5)	133 (12.0)		186	43 (23.1)	44 (23.7)	59 (31.7)	34 (18.3)	6 (3.2)	
Serum Creatinine (mmol/L), n (%)														
Men < 54 or Women < 44	719	281 (39.1)	143 (19.9)	168 (23.4)	89 (12.4)	38 (5.3)	< 0.001	1383	688 (49.8)	311 (22.5)	261 (18.9)	107 (7.7)	16 (1.2)	< 0.001
Men: 54~106 or Women: 44~97	11,715	3978 (34.0)	2862 (24.4)	3143 (26.8)	1431 (12.2)	301 (2.6)		10,461	5325 (50.9)	2339 (22.4)	2085 (19.9)	632 (6.0)	80 (0.8)	
Men > 106 or Women > 97	123	34 (27.6)	35 (28.5)	24 (19.5)	26 (21.1)	4 (3.3)		37	10 (27.0)	11 (29.7)	7 (18.9)	8 (21.6)	1 (2.7)	
Blood Urea Nitrogen (μmol/L), n (%)														
< 2.86	101	36 (35.6)	23 (22.8)	26 (25.7)	12 (11.9)	4 (4.0)	< 0.001	523	300 (57.4)	110 (21.0)	81 (15.5)	30 (5.7)	2 (0.4)	0.027
2.86 ~ 7.14	11,465	3853 (33.6)	2779 (24.2)	3103 (27.1)	1420 (12.4)	310 (2.7)		11,016	5570 (50.6)	2469 (22.4)	2196 (19.9)	690 (6.3)	91 (0.8)	
> 7.14	991	404 (40.8)	238 (24.0)	206 (20.8)	114 (11.5)	29 (2.9)		342	153 (44.7)	82 (24.0)	76 (22.2)	27 (7.9)	4 (1.2)	
Total Cholesterol (mmol/L), n (%)														
< 2.8	82	66 (80.5)	12 (14.6)	3 (3.7)	1 (1.2)	0 (0.0)	< 0.001	44	34 (77.3)	6 (13.6)	4 (9.1)	0 (0.0)	0 (0.0)	< 0.001
2.8 ~ 5.17	7665	3252 (42.4)	1878 (24.5)	1777 (23.2)	671 (8.8)	87 (1.1)		7273	4561 (62.7)	1425 (19.6)	970 (13.3)	287 (4.0)	30 (0.4)	
> 5.17	4810	975 (20.3)	1150 (23.9)	1555 (32.3)	874 (18.2)	256 (5.3)		4564	1428 (31.3)	1230 (27.0)	1379 (30.2)	460 (10.1)	67 (1.5)	
Fasting plasma glucose (mmol/L), n (%)														
< 3.9	20	9 (45.0)	6 (30.0)	2 (10.0)	3 (15.0)	0 (0.0)		20	9 (45.0)	5 (25.0)	3 (15.0)	2 (10.0)	1 (5.0)	< 0.001
3.9 ~	9833	3591 (36.5)	2447 (24.9)	2521 (25.6)	1062 (10.8)	212 (2.2)	< 0.001	10,194	5607 (55.0)	2217 (21.8)	1814 (17.8)	497 (4.9)	59 (0.6)	
6.1 ~	1370	368 (26.9)	309 (22.6)	416 (30.4)	218 (15.9)	59 (4.3)		933	245 (26.3)	263 (28.2)	290 (31.1)	119 (12.8)	16 (1.7)	
> 7.0	1334	325 (24.4)	278 (20.8)	396 (29.7)	263 (19.7)	72 (5.4)		734	162 (22.1)	176 (24.0)	246 (33.5)	129 (17.6)	21 (2.9)	
High Density Lipoprotein Cholesterol (mmol/L), n (%)														
< 0.9	2274	371 (16.3)	465 (20.5)	775 (34.1)	502 (22.1)	161 (7.1)	< 0.001	709	98 (13.8)	134 (18.9)	234 (33.0)	197 (27.8)	46 (6.5)	< 0.001
0.9 ~ 2.19	10,262	3904 (38.0)	2573 (25.1)	2559 (24.9)	1044 (10.2)	182 (1.8)		11,093	5865 (52.9)	2514 (22.7)	2115 (19.1)	548 (4.9)	51 (0.5)	
> 2.19	21	18 (85.7)	2 (9.5)	1 (4.8)	0 (0.0)	0 (0.0)		79	60 (76.0)	13 (16.5)	4 (5.1)	2 (2.5)	0 (0.0)	
Hyperuricemia (men > 440 μmol/L or women > 360 μmol/L)														
No	11,305	4068 (36.0)	2791 (24.7)	2948 (26.1)	1246 (11.0)	252 (2.2)	< 0.001	11,193	5877 (52.5)	2507 (22.4)	2112 (18.9)	614 (5.5)	83 (0.7)	< 0.001
Yes	1252	225 (18.0)	249 (19.9)	387 (30.9)	300 (24.0)	91 (7.3)		688	146 (21.2)	154 (22.4)	241 (35.0)	133 (19.3)	14 (2.0)	

Univariate logistical regression analysis (Model 1) in men showed that the above normal, slightly damaged, moderately damaged and severely damaged TG subgroups had an OR of 1.61 (95% CI: 1.34–1.94), 2.37 (95% CI: 2.00–2.82), 4.35 (95% CI: 3.62–5.23), and 6.53 (95% CI: 4.96–8.59), respectively; age-adjusted logistical regression analysis (Model 2) and multivariate logistical regression model (Model 3) showed a similar trend (Table 2). In all three models, the OR of hyperuricemia was positively associated with an increase in TG (*p* <  0.001). In women, age-adjusted logistic regression analysis showed a similar trend to that of univariate logistic regression model, in accordance with the results in men. Moderately damaged and severely damaged TG subgroups in Model 3 showed an OR of 5.42 (95% CI: 4.03–7.28) and 4.01 (95% CI: 2.13–7.55), respectively. In women, all the three models showed that TG was positively associated with risk for hyperuricemia (all *p* <  0.001).

**Table 2:** Odds ratio of hyperuricemia among fasting plasma triglyceride (mmol/L) in men and women^a^

	Men, fasting plasma triglyceride (mmol/L)						Women, fasting plasma triglyceride (mmol/L)					
	< 1.21	1.21 ~	1.7 ~	2.83 ~	> 5.6	P for trend^b^	< 1.21	1.21 ~	1.7 ~	2.83 ~	> 5.6	P for trend^b^
No. of participants	4293	3040	3335	1546	343		6023	2661	2353	747	97	
Hyperuricemia^c^												
Cases	225	249	387	300	91		146	154	241	133	14	
Rate (%)	5.2	8.2	11.6	19.4	26.5		2.4	5.8	10.2	17.8	14.4	
Model 1, OR (95% CI)	1.00	1.61 (1.34–1.94)	2.37 (2.00–2.82)	4.35 (3.62–5.23)	6.53 (4.96–8.59)	0.000	1.00	2.47 (1.96–3.12)	4.59 (3.72–5.68)	8.72 (6.80–11.19)	6.79 (3.77–12.24)	0.000
Model 2, OR (95% CI)	1.00	1.62 (1.35–1.96)	2.37 (2.00–2.81)	4.26 (3.54–5.12)	6.26 (4.76–8.25)	0.000	1.00	2.35 (1.86–2.97)	4.28 (3.44–5.33)	8.16 (6.33–10.53)	6.43 (3.56–11.61)	0.000
Model 3, OR (95% CI)	1.00	1.46 (1.21–1.77)	1.99 (1.65–2.39)	3.10 (2.51–3.82)	4.27 (3.13–5.83)	0.000	1.00	1.95 (1.53–2.50)	3.20 (2.51–4.08)	5.42 (4.03–7.28)	4.01 (2.13–7.55)	0.000

^a^Logistic regression. Model 1, univariate; Model 2, adjusted with age; Model 3, adjusted with age, alanine aminotransferase (U/L), γ-glutamyl transpeptidase (U/L), serum creatinine (mmol/L), blood urea nitrogen (μmol/L), total cholesterol (mmol/L), plasma glucose (mmol/l), and high density lipoprotein cholesterol (mmol/L). *OR* Odds ratio, *CI* Confidence interval

^b^Contrasts of marginal linear predictions after logistic regression with Stata13

^c^Hyperuricemia:(men > 440 μmol/L or women > 360 μmol/L

Multivariate logistic regression model showed that the positive association between hyperuricemia and TG was significant in men of 20~, 30 ~, 40 ~, 50 ~, 60 ~ and 80 ~ age subgroups (*p* <  0.05, Table 3). Increase in hyperuricemia in the severely damaged TG group was more significant for the 60~ age subgroup (OR = 12.07; 95% CI: 3.63–40.16). In women, the positive association was the lowest in the 50~ age group with increase in TG. No significant increase in hyperuricemia was observed in the 60 ~ (women) and 70 ~ (women and men) age groups (*p* > 0.05).

**Table 3:** Odds ratio of hyperuricemia among fasting plasma triglyceride (mmol/L) and age in men and women^a^

	Men, fasting plasma triglyceride (mmol/L)									Women, fasting plasma triglyceride (mmol/L)								
	No. of participants	Case	Rate (%)	< 1.21	1.21 ~	1.7 ~	2.83 ~	> 5.6	P for trend^b^	No. of participants	Case	Rate (%)	< 1.21	1.21 ~	1.7 ~	2.83 ~	> 5.6	P for trend^b^
No. of participants	12,557	1252	10.0	4293	3040	3335	1546	343		11,881	688	5.8	6023	2661	2353	747	97	
Hyperuricemia^c^																		
< 20 yrs	43	10	23.3	1.00	(−)	(−)	(−)	(−)	(−)	22	3	13.6	1.00	(−)	(−)	(−)	(−)	(−)
20~ yrs	1325	175	13.2	1.00	1.02 (0.63–1.64)	1.50 (0.92–2.45)	2.40 (1.36–4.23)	3.03 (1.11–8.22)	0.005	1880	116	6.2	1.00	2.26 (1.26–4.03)	2.17 (1.13–4.16)	5.98 (2.79–12.83)	7.42 (1.43–38.36)	0.004
30~ yrs	2348	304	12.9	1.00	1.70 (1.11–2.59)	1.78 (1.18–2.70)	3.10 (1.95–4.91)	3.88 (2.06–7.30)	0.000	2485	107	4.3	1.00	3.01 (1.74–5.20)	3.22 (1.75–5.90)	6.64 (3.11–14.16)	8.10 (2.10–31.17)	0.000
40~ yrs	3133	288	9.2	1.00	1.80 (1.10–2.93)	3.22 (2.06–5.02)	4.92 (3.03–7.99)	6.73 (3.58–12.68)	0.000	2652	74	2.8	1.00	3.48 (1.76–6.86)	3.95 (1.91–8.18)	6.13 (2.40–15.64)	(−)	0.001
50~ yrs	2735	241	8.8	1.00	1.38 (0.88–2.15)	2.04 (1.34–3.11)	4.07 (2.57–6.45)	7.48 (3.77–14.85)	0.000	2492	159	6.4	1.00	1.58 (0.94–2.66)	2.31 (1.38–3.86)	4.12 (2.26–7.49)	5.07 (1.71–15.00)	0.000
60~ yrs	1848	123	6.7	1.00	2.20 (1.16–4.15)	4.17 (2.29–7.59)	5.43 (2.57–11.45)	12.07 (3.63–40.16)	0.000	1509	123	8.2	1.00	0.82 (0.38–1.76)	3.79 (1.99–7.22)	5.59 (2.64–11.85)	1.15 (0.13–10.08)	0.549
70~ yrs	848	81	9.6	1.00	1.12 (0.58–2.16)	1.07 (0.51–2.25)	1.48 (0.55–3.99)	4.46 (0.70–28.41)	0.096	679	78	11.5	1.00	1.53 (0.68–3.41)	2.82 (1.34–5.97)	1.06 (0.33–3.40)	(−)	0.570
80~ yrs	277	30	10.8	1.00	4.80 (1.58–14.56)	5.04 (1.26–20.19)	10.13 (1.58–64.80)	(−)	0.018	162	28	17.3	1.00	1.70 (0.32–8.87)	3.75 (0.71–19.67)	16.75 (2.26–124.18)	(−)	0.006

^a^Logistic regression model adjusted with alanine aminotransferase (U/L), γ-glutamyl transpeptidase (U/L), serum creatinine (mmol/L), blood urea nitrogen (μmol/L), total cholesterol (mmol/L), plasma glucose (mmol/L), and high density lipoprotein cholesterol (mmol/L)

^b^Contrasts of marginal linear predictions from 3.9~ though > 7.0 groups after logistic regression with Stata13

^c^Hyperuricemia:(men > 440 μmol/L or women > 360 μmol/L

Considering the complicated relationship between triglyceride and glucose metabolism, we also analyzed the association of triglyceride-glucose index (TyG) with hyperuricemia. The results showed that TyG tended to be a better index of hyperuricemia in females than in males (Table 4). After being stratified by the age, the better behavior of TyG was concentrated in 40~ and 60~ age groups (Table 5).

**Table 4:** Odds ratio of hyperuricemia among triglyceride-glucose index in men and women^a^

	Men, triglyceride-glucose index (TyG)					Women, triglyceride-glucose index (TyG)				
	0 ~	1st quatile ~	2nd quatile ~	3rd quatile ~	P for trend^b^	0 ~	1st quatile ~	2nd quatile ~	3rd quatile ~	P for trend^b^
No. of participants	2169	3034	3410	3944		3924	3057	2701	2199	
Hyperuricemia^c^										
Cases	103	199	333	617		67	114	198	309	
Rate (%)	4.7	6.6	9.8	15.6		1.7	3.7	7.3	14.1	
Model 1, OR (95% CI)	1.00	1.40 (1.10–1.79)	2.16 (1.72–2.72)	3.72 (3.00–4.62)	< 0.001	1.00	2.23 (1.64–3.33)	4.53 (3.41–6.71)	9.41 (7.19–12.33)	< 0.001
Model 2, OR (95% CI)	1.00	1.47 (1.15–1.88)	2.26 (1.80–2.85)	3.90 (3.14–4.84)	< 0.001	1.00	2.19 (1.61–2.98)	4.39 (3.29–5.86)	9.08 (6.85–12.72)	< 0.001
Model 3, OR (95% CI)	1.00	1.32 (1.03–1.72)	1.85 (1.46–2.35)	2.68 (2.11–3.41)	< 0.001	1.00	1.90 (1.39–2.61)	3.32 (2.44–4.52)	6.08 (4.43–8.34)	< 0.001

^a^Logistic regression. Model 1, univariate; Model 2, adjusted with age; Model 3, adjusted with age, alanine aminotransferase (U/L), γ-glutamyl transpeptidase (U/L), serum creatinine (mmol/L), blood urea nitrogen (μmol/L), total cholesterol (mmol/L), and high density lipoprotein cholesterol (mmol/L). *OR* Odds ratio, *CI* Confidence interval

^b^Contrasts of marginal linear predictions after logistic regression with Stata13

^c^Hyperuricemia:(men > 440 μmol/L or women > 360 μmol/L

**Table 5:** Odds ratio of hyperuricemia among triglyceride-glucose index and age in men and women^a^

	Men, triglyceride-glucose index (TyG)								Women, triglyceride-glucose index (TyG)							
	No. of participants	Case	Rate (%)	0 ~	1st quatile ~	2nd quatile ~	3rd quatile ~	P for trend^b^	No. of participants	Case	Rate (%)	0 ~	1st quatile ~	2nd quatile ~	3rd quatile ~	P for trend^b^
No. of participants	12,557	1252	10.0	2169	3034	3410	3944		11,881	688	5.8	3924	3057	2701	2199	
Hyperuricemia^c^																
< 20 yrs	43	10	23.3	1.00	4.98 (0.37–66.42)	73.31 (1.18–4529.68)	7.78 (0.20–297.59)	0.135	22	3	13.6	1.00	(−)	(−)	(−)	(−)
20~ yrs	1325	175	13.2	1.00	1.19 (0.70–2.77)	1.29 (0.76–2.18)	2.44 (1.41–4.24)	0.002	1880	116	6.2	1.00	2.77 (1.50–5.97)	2.49 (1.23–5.23)	5.72 (2.82–11.61)	< 0.001
30~ yrs	2348	304	12.9	1.00	1.30 (0.77–2.21)	1.83 (1.11–3.32)	2.27 (1.36–3.78)	< 0.001	2485	107	4.3	1.00	1.89 (0.96–3.69)	4.15 (2.18–7.89)	5.95 (2.92–12.11)	< 0.000
40~ yrs	3133	288	9.2	1.00	0.98 (0.51–1.89)	2.03 (1.13–3.65)	3.77 (2.12–6.72)	< 0.001	2652	74	2.8	1.00	3.61 (1.29–13.86)	9.42 (3.42–25.97)	11.42 (3.87–33.73)	< 0.001
50~ yrs	2735	241	8.8	1.00	1.54 (0.81–2.93)	1.92 (1.04–3.54)	3.31 (1.80–6.75)	< 0.001	2492	159	6.4	1.00	0.91 (0.46–1.79)	1.64 (0.87–3.57)	2.86 (1.51–5.42)	0.002
60~ yrs	1848	123	6.7	1.00	3.17 (1.06–9.46)	4.90 (1.67–14.37)	6.46 (2.19–19.53)	< 0.001	1509	123	8.2	1.00	2.27 (0.62–8.31)	3.98 (1.18–13.47)	10.67 (3.17–35.98)	< 0.001
70~ yrs	848	81	9.6	1.00	1.48 (0.62–3.54)	1.35 (0.54–3.36)	1.34 (0.53–3.41)	0.600	679	78	11.5	1.00	0.97 (0.30–3.14)	1.60 (0.53–4.78)	2.25 (0.74–6.83)	0.198
80~ yrs	277	30	10.8	1.00	1.39 (0.29–6.72)	6.16 (1.51–25.72)	3.68 (0.72–18.71)	0.035	162	28	17.3	(−)	1.00	3.08 (0.67–14.27)	5.24 (1.10–24.87)	0.321

^a^Logistic regression model adjusted with alanine aminotransferase (U/L), γ-glutamyl transpeptidase (U/L), serum creatinine (mmol/L), blood urea nitrogen (μmol/L), total cholesterol (mmol/L), and high density lipoprotein cholesterol (mmol/L)

^b^Contrasts of marginal linear predictions from 3.9~ though > 7.0 groups after logistic regression with Stata13

^c^Hyperuricemia: men > 440 μmol/L or women > 360 μmol/L

## Discussion

In this study, we found a positive relationship of high triglyceride with hyperuricemia in both men and women, in agreement with an Indian study [14]. Dietary habits have been changing in both China and India, and obesity and hypertriglyceridemia have begun to occur in younger people.

The relationship of serum uric acid with triglyceridemia is still controversial. Earlier studies showed that hyperuricemia was always accompanied with hypertriglyceridemia initially [15, 16]. In addition, TG was more strongly associated with serum UA than HDL-C and TC, using the healthy examination data [17]. A Kuwait study further supported the close relation in dyslipidemic patients, a group already at high coronary artery disease risk [18]. Nevertheless, whether age and gender differences mediate the association between serum UA levels and TG is still controversial. In present study, we demonstrated that after adjustment with age (Model 2) and other confounding factors (Model 3), the OR still increased with the increase in TG. These results suggest that TG levels independently affect the incidence of hyperuricemia. However, in multivariate logistic regression analysis, the positive association between hyperuricemia and TG showed a gender and age differences and the positive association was the lowest in the 50~ age group. Whether decreasing level of estrogen after menopause is responsible for the differences needs further studies.

Stelmach et al. investigated 607 Polish adults with hyperuricemia and demonstrated that the upper tertile of serum uric acid levels had higher TG values in males but not in females [19]. In contrast, Lippi et al. retrospectively enrolled a large cohort of unselected adult outpatients and showed that triglycerides were independently associated with serum UA in women but not in men [20]. Notably, in this study our data showed that high TG level was positively associated with the incidence of hyperuricemia in both men and women. This is consistent with a prospective study which demonstrated that hypertriglyceridemia in men might strengthen the effect of serum UA on the development of gout [21]. Chinese diet is characterized with a high-fat diet, particularly the overconsumption of cooking oil may be a significant risk factor for obesity [22, 23].

To investigate lipid abnormalities in acute myocardial infarction (AMI) patients, Wei et al. retrospectively analyzed 1213 AMI patients in East China and showed a significant difference in triglycerides for male but not for female AMI patients [24]. Xu et al. found that older Chinese people had moderate and high levels of unbalanced diets [25]. Significant differences were influenced by many factors, such as gender, marital status, work status, education levels. These findings highlight complex interaction between hyperuricemia and TG. Differences in dietary patterns such as the proportion of carbohydrate or fat may be responsible for the variability in the relationship between serum uric acid and triglyceridemia.

TyG index is proposed as a marker of moderate insulin resistance. Therefore, we analyzed the association of TyG with hyperuricemia. We found that TyG could be a better index of hyperuricemia in females than in males. However, a recent study indicated that TyG index presented the significant risks for chronic kidney disease in both men and women [26]. The reason for the disparities is unclear and need additional investigations.

Our study has two main limitations. First, our study was conducted in a special group, so the generalizability of our findings to other population needs confirmation in future studies. Second, confounding factors such as diet patterns and health concerns among people of different ages were not included in our analysis, which may have an impact on the results. Further studies are required to elucidate the association between triglyceride and hyperuricemia in different gender and age groups.

## Conclusions

Our study demonstrated that high triglyceride was positively associated with hyperuricemia in both men and women, and this association was age-related, especially in women.

## Data Availability

All data and material are available upon request.

## Abbreviations

ALT: Alanine aminotransferase
BUN: Blood urea nitrogen
HDL-C: High-density lipoprotein cholesterol
SCr: Serum creatinine
TC: Total cholesterol
TG: Triglyceride
UA: Uric acid
γ-GT: γ-glutamyl transpeptidase
